# Selection and evaluation of bi-allelic autosomal SNP markers for paternity testing in Koreans

**DOI:** 10.1007/s00414-020-02495-7

**Published:** 2021-04-28

**Authors:** Soyeon Bae, Sohyoung Won, Heebal Kim

**Affiliations:** 1 Department of Agricultural Biotechnology and Research Institute of Agriculture and Life Sciences, Seoul National University, Seoul, 08826 Republic of Korea; 2 Interdisciplinary Program in Bioinformatics, Seoul National University, Seoul, 08826 Republic of Korea; 3 eGnome, Inc., Seoul, Republic of Korea

**Keywords:** Single-nucleotide polymorphism, Paternity testing, Korean, False inclusion

## Abstract

Due to the advantages of single-nucleotide polymorphisms (SNPs) in forensic science, many forensic SNP panels have been developed. However, the existing SNP panels have a problem that they do not reflect allele frequencies in Koreans or the number of markers is not sufficient to perform paternity testing. Here, we filtered candidate SNPs from the Ansan-Ansung cohort data and selected 200 SNPs with high allele frequencies. To reduce the risk of false inclusion and false exclusion, we calculated likelihood ratios of alleged father-child pairs from simulated families when the alleged father is the true father, the close relative of the true father, and the random man. As a result, we estimated that 160 SNPs were needed to perform paternity testing. Furthermore, we performed validation using Twin-Family cohort data. When 160 selected SNPs were used to calculate the likelihood ratio, paternity and non-paternity were accurately distinguished. Our set of 160 SNPs could be useful for paternity testing in Koreans.

**Supplementary Information:**

The online version contains supplementary material available at 10.1007/s00414-020-02495-7.

## Introduction

In modern forensic science, DNA profiling has become an important tool for human identification and paternity testing. Short tandem repeat (STR) markers, usually composed of 13-17 loci, and recently expanded to 21 or more loci, have generally been used for DNA profiling [1–3]. However, advances in sequencing technologies have enabled the production of large amounts of single-nucleotide polymorphism (SNP) data, and this led to a discussion about the availability of SNP markers in the field of forensic science.

Compared to STRs, SNP markers have the advantage of low mutation rate, small amplicon size, which is advantageous for analysis of degraded samples, and fast and automated analysis [4, 5]. On the other hand, more SNPs are needed to approach the match probability of STR panels since bi-allelic SNPs are less polymorphic than STRs. Krawczak [6] and Gill [7] reported that 50-60 SNPs with allele frequencies close to 0.5 are required to have the same discriminatory power as STR panels. Ayres [8] suggested that the number of SNPs with allele frequencies in the range [0.3, 0.5] required for the standard trio (father-mother-child) case and duo (father-child) case is 50-60 and 70-80, respectively. However, these studies assumed the use of independent markers. When the number of markers increases, the probability of genetic linkage increases. Since the use of markers that are not independently transmitted can affect the results of the forensic analysis, linkage should be considered in forensic calculations [9, 10].

Several bi-allelic autosomal marker panels, such as the SNPforID multiplex (52 SNPs) [11] and the IISNP panel (86 SNPs) [12–14], were developed for human identification and paternity testing. However, if the alleged father (AF) is the close relative of the true father (TF), there may be cases where the number of SNP loci used in existing panels is not enough to perform paternity testing [15]. In addition, these panels were selected based on allele frequencies of various human populations. As allele frequencies may vary by population, markers selected based on allele frequencies of a certain population may not sufficiently reflect allele frequencies of another population. Paternity testing using incorrect allele frequencies can lead to erroneous results [16, 17]. Thus, several studies have developed forensic SNP panels for a specific population [18, 19]. Lee et al. [20] and Kim et al. [21] selected highly informative SNPs from Koreans for forensic purposes and provided a database, but the number of markers was 24 and 30, respectively, which was insufficient to perform paternity testing.

In this study, we aimed to select bi-allelic autosomal SNP markers for paternity testing for Korean individuals based on likelihood ratio (LR) principles, where genetic evidence is evaluated by calculating the LR [22]. Korean SNP data were screened to collect candidate markers. Allele frequencies of retained SNPs were calculated, and based on this information, we selected the appropriate number of markers using simulated family data. Moreover, we examined the performance of final set of SNPs in real cases.

## Materials and methods

### Sample collection

We used SNP genotyping data from the Ansan-Ansung cohort and the Twin-Family cohort, which were part of the Korean Genome and Epidemiology Study (KoGES) [23]. DNA was extracted from blood samples collected from individuals. The participants of the Ansan-Ansung cohort study were 10,030 adults aged 40 to 69, who live in Ansan or Ansung. Among them, 8840 individuals were genotyped with the Affymetrix Genome-Wide Human SNP Array 5.0. The Twin-Family cohort study, consisting of 3202 twins and their families, collected SNP data from 1716 individuals using the Affymetrix Genome-Wide Human SNP Array 6.0.

### Quality control and SNP selection

In this study, the selection of candidate SNP markers and calculation of allele frequencies were based on the Ansan-Ansung cohort data, and the performance of markers was evaluated using the Twin-Family cohort data. The Ansan-Ansung cohort data included 352,228 bi-allelic autosomal SNPs. Among them, SNPs not included in the Twin-Family cohort data were excluded. To avoid the influence of selection pressure, SNPs within the range of a gene list (hg19) were discarded. Quality control (QC) steps were performed as follows: (1) Samples with individual missing rates higher than 0.05 were filtered. (2) SNPs with missing genotype rates higher than 0.01 were removed. (3) SNPs that deviated from the Hardy-Weinberg equilibrium (*p* value < 10^−5^) were removed. Next, we estimated kinship coefficients to identify potential relatives. Samples were filtered until kinship coefficients of all pairs of individuals were lower than 0.0884, meaning that all pairs were treated as third-degree or more distant relationships. The fixation index (*F*_ST_) was calculated between Ansan and Ansung populations. Then, we performed linkage disequilibrium (LD)-based SNP pruning with the following parameters: window size = 500, step size = 50, and *r*^2^ threshold = 0.01. Finally, 200 candidate SNPs with the highest minor allele frequencies (MAFs) were selected among the retained SNPs. The minimum distance between candidate SNPs in each chromosome was 10 Mbp. PLINK v1.90 was used to conduct QC steps and LD pruning and to calculate *F*_ST_ and MAF [24]. Kinship coefficient was estimated using KING v2.2.4 [25].

### Testing in simulated pedigrees

**Fig. 1:**
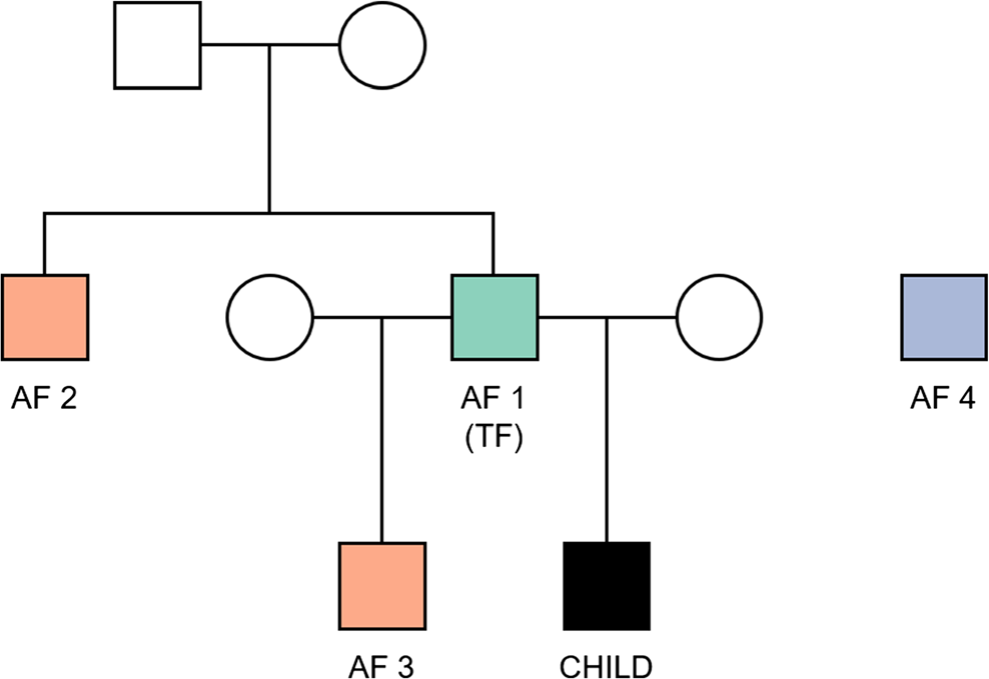
Structure of simulated pedigrees (AF 1: TF, AF 2: brother of TF, AF 3: child of TF, AF 4: random man)

In order to select the appropriate number of markers needed for the paternity test, we simulated 10,000 pedigrees using MERLIN v1.1.2 [26]. Since this program requires centimorgan (cM) position for each SNP, we obtained this information from the genetic map of the CHB (Han Chinese in Beijing, China) population of the 1000 Genomes Project (available at ftp://ftp.1000genomes.ebi.ac.uk/vol1/ftp/technical/working/20130507_omni_recombination_rates/). If there was no information about the genetic position of the SNP, the genetic position of the nearest SNP was used. The structure of simulated pedigrees is shown in Fig. 1. Founder genotypes were randomly generated based on the previously calculated allele frequencies of candidate SNPs. Then, it was assumed that each parent contributes one allele to the offspring. Alleles spaced less than 25 cM were clustered and passed to the offspring based on the estimated haplotype frequencies.

In paternity testing, we evaluated genetic evidence by comparing likelihoods of hypotheses using the equation: LR = Pr(*G*|*H*_parent-child_)/Pr(*G*|*H*_unrelated_), where *G* is the observed genotype data, *H*_parent-child_ is the hypothesis that two tested people are in a parent-child relationship, and *H*_unrelated_ is the hypothesis that two tested people are unrelated [22]. We calculated LRs for each marker and multiplied them all to obtain final LRs for a set of markers using a “likelihoodMerlin” function of “pedprobr” package in R [26, 27]. This function can take into account linkage when calculating LRs. Ayres [8] estimated that at least 70 SNP loci were required for paternity testing in the motherless duo case, so the number of markers used in the analysis was increased from 70 to 200 by 10. When the log_10_LR was greater than or equal to 5, which provides very strong support for *H*_parent-child_ [9], AF and child were judged to be in a parent-child relationship. The accuracy is defined as the percentage by which the relationship of two individuals is correctly determined as parent-child or non-parent-child. The false positive is an error in which a person who is not the TF is falsely included as the TF. In contrast, the false negative means that the TF is falsely excluded.

### Validation in real cases

The Twin-Family cohort data was used to examine the performance of selected markers. Individuals with missing genotype data of these SNPs were filtered out. We collected all pairs of two individuals who were in parent-child or unrelated relationships. We also used second-degree relative pairs, such as uncle-nephew or grandparent-grandchild, to check LR values when the AF is the close relative of the TF. The methods used to decide paternity and calculate the accuracy, false positive, and false negative were written above. The method of maternity testing was the same as that of paternity testing, except that only the gender of the typed person is female. Thus, we calculated LRs for all pairs of individuals, ignoring their gender.

## Results

### Candidate SNP selection

The number of bi-allelic autosomal SNPs included in both data was 280,905. Since functional markers have a possibility that the selection pressure affects the allele frequency [9], 125,485 SNPs in the gene region were excluded to avoid this. A total of 12,238 SNPs with a genotyping rate lower than 0.99 were removed. The total genotyping rate of the retained SNPs from the Ansan-Ansung cohort was 0.9987. One hundred ninety-three SNPs were removed due to failure to pass the Hardy-Weinberg exact test. Among 8840 participants with a genotyping rate per individual higher than 0.95, 8621 unrelated samples were selected. All SNPs had *F*_ST_ values lower than 0.01, so it could be considered that there was no significant genetic difference between the populations in these regions. Finally, 10,538 independent (pairwise *r*^2^ < 0.01) SNPs were retained by the LD pruning method.

To select highly informative SNPs, we calculated MAFs of 10,538 SNPs from 8621 unrelated samples and selected 200 SNPs located far from each other (> 10 Mbp) with a high MAF. These SNPs had an MAF in the range [0.49, 0.5].

### Paternity testing in simulated pedigrees

A total of 10,000 families were generated to determine the appropriate number of markers for paternity testing. Within each family, we were able to collect four types of AF-child pairs as shown in Fig. 1: The AF was the TF (AF 1), the AF was the close relative of TF (AF 2 and 3), and the AF was the unrelated person (AF 4). LRs were calculated for each pair, using 70, 80, …, and 200 SNPs.

Table 1 shows the accuracy, false-positive rate, and false-negative rate in simulated duo cases. Since we used a high value of LR cutoff, the false-negative rate was very high with 70-80 loci, which were suggested in a previous study [8]. When the number of loci was 150, no TF was falsely excluded. However, there were some cases that the first-degree relative of the TF was judged to be the TF, so the false-positive rate was 0.0033%. One hundred percent accuracy was achieved when 160 or more SNPs were used. As a result, we selected a set of 160 SNPs for paternity testing without errors. The details of the selected SNPs are shown in Supplementary Table 1.

**Table 1:** Summary of simulated results

Number of markers	Accuracy (%)	False-positive rate (%)	False-negative rate (%)
70	89.72	0.18	40.58
80	95.0725	0.22	19.05
90	98.0975	0.17	7.1
100	99.3625	0.1033	2.24
110	99.7825	0.08333	0.62
120	99.945	0.03333	0.12
130	99.975	0.01667	0.05
140	99.9925	0.006667	0.01
150	99.9975	0.003333	0
160 ≤	100	0	0

**Fig. 2:**
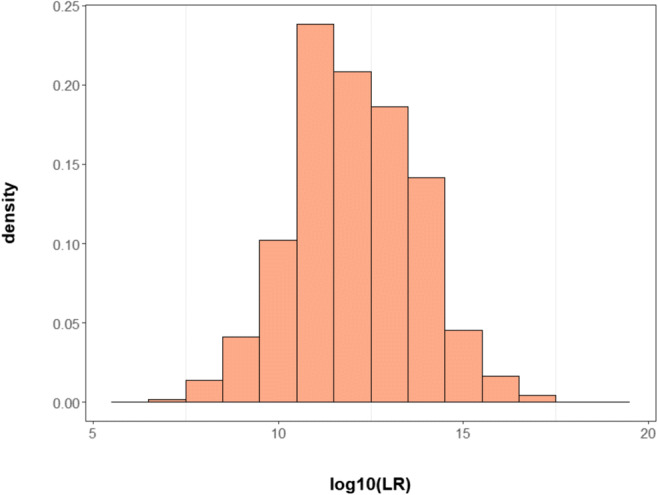
Distribution of log_10_LR values for true parent-child pairs in simulation results using 160 SNPs

Figure 2 shows the distribution of log_10_LR values for true parent-child pairs. The average, minimum, and maximum values of the log_10_LR for parent-child pairs were 12.05, 6.02, and 18.98, respectively. In non-parent-child pairs, LRs were all zero due to SNP mismatches between the two tested people.

### Paternity testing in real cases

**Fig. 3:**
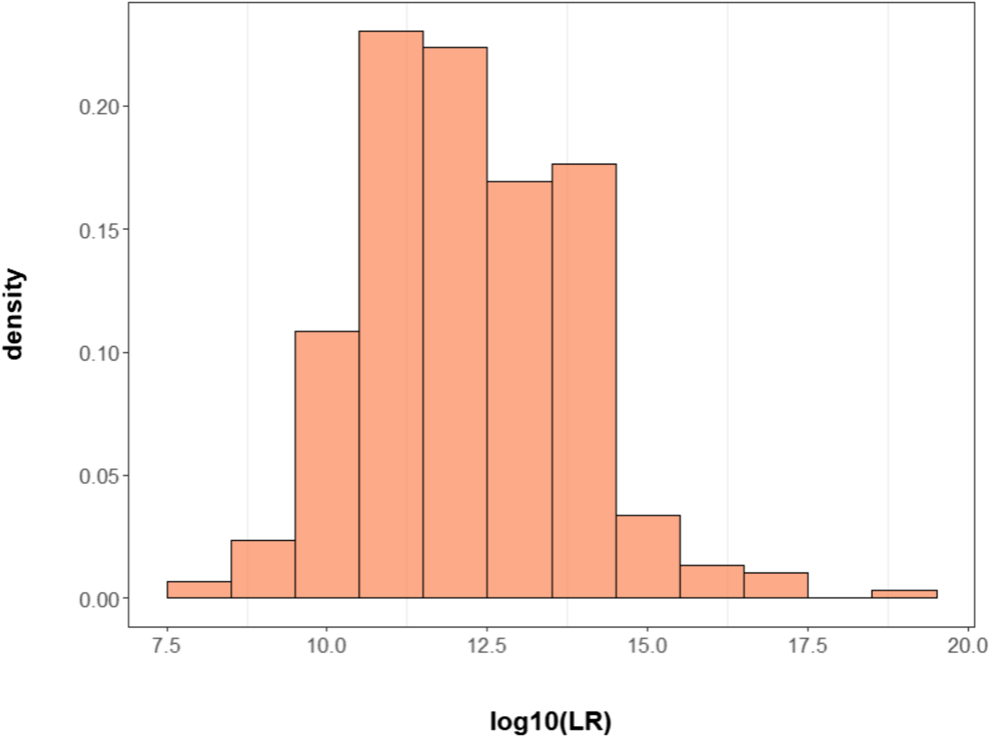
Distributions of log_10_LR values for true parent-child pairs in real cases using 160 SNPs

The family genotype data included 1716 samples. Among them, 816 samples, whose genotypes of finally selected SNPs were observed, were used for validation. Out of a total of 332,520 pairs, those with unknown or uncertain relationships were excluded from the results. Finally, there were 295, 19, and 331,778 pairs of parent-child, second-degree relative, and unrelated individuals, respectively. When paternity testing was performed using 160 selected SNPs, the accuracy reached 100%. The LR values for non-parent-child pairs were zero, and the average, minimum, and maximum values of the log_10_LR for parent-child pairs were 12.18, 8.42, and 19.26, respectively (Fig. 3). We also identified the number of opposite homozygosity for 160 SNPs, which means the child and the AF are homozygous and have different alleles; for example, the child has allele AA and the AF has allele BB [15, 28]. The more mismatches are observed, the less likely the two individuals are in parent-child relationships. No mismatch was found in any of the 160 loci in all true parent-child relationships. When the AF was the first-degree relative of the TF and the unrelated man, the average number of mismatches between the AF and the child was 10.95 and 20, respectively.

## Discussion

After the usefulness of SNP-based human identification and paternity testing was discussed, several sets of forensic SNP markers were developed. SNPforID [11] and IISNP [14] are universal forensic SNP panels for various populations. However, SNPforID panel consists of 52 loci, which is an insufficient number of markers to perform paternity testing of duo cases [8]. Børsting et al. [28] observed that false association occurred in some duo cases when using SNPforID panel. In addition, according to NCBI dbSNP (https://www.ncbi.nlm.nih.gov/snp/), 19 and 7 SNPs in SNPforID and IISNP, respectively, had an MAF value lower than 0.3 in East Asians based on 1000 Genomes Project data. SNPs with a low MAF are less informative and may not be the best choice for forensic analysis in East Asians (Supplementary Fig. 1). Furthermore, since there are various populations within East Asians, it is unclear whether the existing allele frequency database is accurate for Koreans. It is important to accurately estimate allele frequencies of the population to reduce errors in forensic analysis [16, 17]. Although several studies have selected forensic SNP marker sets for Koreans and provided allele frequency information [20, 21], these panels are expected to be unsuitable for paternity testing because they consist of fewer than 50 SNPs, which are suggested to be needed for the analysis of trio cases [8].

In the present study, we selected and tested the appropriate number of bi-allelic autosomal markers for paternity testing in Korean individuals. We considered difficult cases when choosing the number of markers. There were special cases where false inclusion occurred when the TF was a close relative of the AF [29, 30]. These problems were solved by supplementing additional markers [31, 32]. We aimed to solve these problems with only autosomal SNPs by selecting a sufficient number of loci and focus on the duo case because there are special cases where genotype of one of the parents is not available.

Of 352,228 SNPs, 200 candidates were selected from 8621 unrelated Korean samples after filtering processes. These markers were non-functional, and had a high MAF (≥ 0.49) and an *F*_ST_ (< 0.01) value between Ansan and Ansung. To minimize the effects of genetic linkage and LD, we selected only SNPs located far from each other with a low level of LD (*r*^2^ < 0.01) between different loci in the population. However, it was still not far enough to assume that these markers were transmitted independently. Thus, we calculated LRs by considering genetic distances from the genetic map of the East Asian population (Han Chinese in Beijing, China). Based on allele frequencies and genetic positions of 200 candidate SNPs, we randomly generated 10,000 families and calculated the LR for parentage. Based on our simulation results, we finally selected highly informative 160 SNP loci to remove falsely included cases. Using these final set of 160 SNPs, all 332,092 comparisons in real cases were determined for paternity and non-paternity.

In summary, we selected 160 SNPs for paternity testing based on allele frequencies in Koreans. Our study showed that using 160 autosomal SNPs with an MAF close to 0.5 in paternity testing would be sufficient to remove the risk of false inclusion. Considering that SNP has a lower mutation rate, which reduces the probability of false exclusion, our final set of SNPs seems to be useful for paternity testing.

## Supplementary information

ESM 1 (DOCX 723 kb)

ESM 2 (DOCX 84 kb)

## Data Availability

The datasets analyzed during the current study are available on request from the National Institute of Health, http://www.nih.go.kr/contents.es?mid=a50401010100.
