# Supplementary material for: Selection and evaluation of bi-allelic autosomal SNP markers for paternity testing in Koreans
Source: Int J Legal Med. 2021 Apr 28;135(4):1369–74. doi: 10.1007/s00414-020-02495-7 (PMC8205914; doi:10.1007/s00414-020-02495-7)
Supplement: Supplementary file 1 — (DOCX 723 kb) [file 414_2020_2495_MOESM1_ESM.docx]

**Selection and evaluation of bi-allelic autosomal SNP markers for paternity testing in Koreans**

Soyeon Bae^1^, Sohyoung Won^2^, Heebal Kim^1,2,3,*^

^1^Department of Agricultural Biotechnology and Research Institute of Agriculture and Life Sciences, Seoul National University, Seoul 08826, Republic of Korea.

^2^Interdisciplinary Program in Bioinformatics, Seoul National University, Seoul 08826, Republic of Korea.

^3^eGnome, Inc, Seoul, Republic of Korea

^*^Corresponding Author: Heebal Kim [heebal@snu.ac.kr](mailto:heebal@snu.ac.kr)


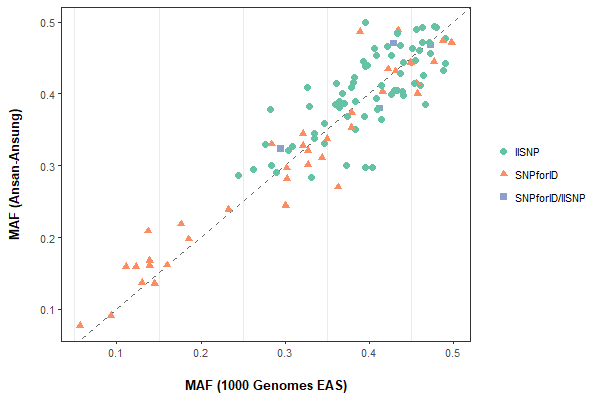


Supplementary Fig. 1 Minor allele frequencies of universal forensic SNPs calculated from East Asians in the 1000 Genomes Project available from NCBI dbSNP and the Ansan-Ansung cohort. Of the total 134 SNPs, only 112 SNPs found in the Ansan-Ansung cohort data are shown
