# Supplementary material for: Selection and evaluation of bi-allelic autosomal SNP markers for paternity testing in Koreans
Source: Int J Legal Med. 2021 Apr 28;135(4):1369–74. doi: 10.1007/s00414-020-02495-7 (PMC8205914; doi:10.1007/s00414-020-02495-7)
Supplement: Supplementary file 2 — (DOCX 84 kb) [file 414_2020_2495_MOESM2_ESM.docx]

**Selection and evaluation of bi-allelic autosomal SNP markers for paternity testing in Koreans**

Soyeon Bae^1^, Sohyoung Won^2^, Heebal Kim^1,2,3,*^

^1^Department of Agricultural Biotechnology and Research Institute of Agriculture and Life Sciences, Seoul National University, Seoul 08826, Republic of Korea.

^2^Interdisciplinary Program in Bioinformatics, Seoul National University, Seoul 08826, Republic of Korea.

^3^eGnome, Inc, Seoul, Republic of Korea

^*^Corresponding Author: Heebal Kim [heebal@snu.ac.kr](mailto:heebal@snu.ac.kr)

| **No** | **Chromosome** | **SNP** | **Genetic position (cM)** | **Alleles** | **MAF** |
| --- | --- | --- | --- | --- | --- |
| 1 | 1 | rs780606 | 6.86 | C/T | 0.4999 |
| 2 | 1 | rs6668468 | 36.98 | G/A | 0.4992 |
| 3 | 1 | rs504242 | 61.29 | G/C | 0.4992 |
| 4 | 1 | rs11206160 | 67.97 | C/G | 0.4998 |
| 5 | 1 | rs1155542 | 84.36 | G/A | 0.4984 |
| 6 | 1 | rs4847319 | 110.72 | C/G | 0.4999 |
| 7 | 1 | rs360603 | 128.75 | G/A | 0.4982 |
| 8 | 1 | rs3811411 | 134.39 | C/A | 0.4984 |
| 9 | 1 | rs1451606 | 152.88 | G/A | 0.4975 |
| 10 | 1 | rs7534794 | 175.32 | G/A | 0.5 |
| 11 | 1 | rs1775151 | 188.48 | C/T | 0.4998 |
| 12 | 1 | rs645142 | 202.94 | C/G | 0.4986 |
| 13 | 1 | rs16857963 | 215.1 | C/G | 0.4994 |
| 14 | 1 | rs10926819 | 237.88 | A/G | 0.4985 |
| 15 | 2 | rs940516 | 8.25 | A/T | 0.4979 |
| 16 | 2 | rs4670906 | 46.28 | C/T | 0.4994 |
| 17 | 2 | rs281493 | 61.92 | C/A | 0.4985 |
| 18 | 2 | rs10179027 | 72 | A/T | 0.4999 |
| 19 | 2 | rs6546815 | 86.32 | A/T | 0.499 |
| 20 | 2 | rs4616480 | 98.21 | T/C | 0.4984 |
| 21 | 2 | rs2723152 | 109.8 | A/G | 0.4992 |
| 22 | 2 | rs13034215 | 118.78 | T/C | 0.499 |
| 23 | 2 | rs7607239 | 131.61 | A/G | 0.4998 |
| 24 | 2 | rs1533586 | 141.21 | G/C | 0.4977 |
| 25 | 2 | rs12693239 | 166.63 | G/A | 0.4999 |
| 26 | 2 | rs4234081 | 177.86 | T/C | 0.4995 |
| 27 | 2 | rs977984 | 201.71 | G/T | 0.4999 |
| 28 | 2 | rs10167210 | 228.4 | C/A | 0.4986 |
| 29 | 3 | rs12492635 | 8.23 | G/A | 0.4992 |
| 30 | 3 | rs6762167 | 33.57 | A/T | 0.4987 |
| 31 | 3 | rs7637245 | 52.88 | C/G | 0.4994 |
| 32 | 3 | rs6776728 | 62.83 | G/A | 0.4992 |
| 33 | 3 | rs753819 | 73.79 | A/G | 0.4994 |
| 34 | 3 | rs17752319 | 95.91 | G/C | 0.4983 |
| 35 | 3 | rs9882970 | 104.1 | C/T | 0.4992 |
| 36 | 3 | rs2712470 | 111.33 | A/G | 0.4991 |
| 37 | 3 | rs6788352 | 121.41 | C/G | 0.4994 |
| 38 | 3 | rs7630727 | 132.32 | C/T | 0.4991 |
| 39 | 3 | rs11915439 | 149.13 | C/T | 0.4999 |
| 40 | 3 | rs901812 | 162.84 | C/T | 0.4988 |
| 41 | 3 | rs16859509 | 173.68 | C/T | 0.4987 |
| 42 | 3 | rs9290953 | 199.65 | C/T | 0.4986 |
| 43 | 4 | rs1402038 | 21.53 | G/A | 0.4999 |
| 44 | 4 | rs7686265 | 37.83 | G/A | 0.5 |
| 45 | 4 | rs2060028 | 73.42 | C/T | 0.4994 |
| 46 | 4 | rs1056079 | 103.51 | C/A | 0.4988 |
| 47 | 4 | rs3114040 | 117.34 | C/T | 0.4991 |
| 48 | 4 | rs893232 | 133.56 | C/T | 0.4999 |
| 49 | 4 | rs12651345 | 188.79 | G/A | 0.4983 |
| 50 | 5 | rs178328 | 7.26 | A/G | 0.4997 |
| 51 | 5 | rs162859 | 29.32 | T/C | 0.4988 |
| 52 | 5 | rs10067897 | 47.12 | C/G | 0.4992 |
| 53 | 5 | rs251529 | 56.94 | G/A | 0.4998 |
| 54 | 5 | rs407455 | 67.25 | A/G | 0.4997 |
| 55 | 5 | rs3909481 | 88.29 | G/A | 0.4988 |
| 56 | 5 | rs6879586 | 94.97 | C/T | 0.4996 |
| 57 | 5 | rs991601 | 103.23 | G/A | 0.4997 |
| 58 | 5 | rs6868806 | 120 | A/T | 0.4995 |
| 59 | 5 | rs17055814 | 146.33 | C/T | 0.4997 |
| 60 | 5 | rs265974 | 175.43 | G/A | 0.4998 |
| 61 | 6 | rs7751978 | 19.54 | T/C | 0.4999 |
| 62 | 6 | rs1611637 | 45.36 | C/T | 0.4994 |
| 63 | 6 | rs3957243 | 56.97 | T/C | 0.4976 |
| 64 | 6 | rs1937142 | 68.75 | C/T | 0.4994 |
| 65 | 6 | rs16900688 | 76.07 | G/A | 0.4999 |
| 66 | 6 | rs6454487 | 85 | G/A | 0.4983 |
| 67 | 6 | rs6917201 | 95.6 | T/C | 0.4999 |
| 68 | 6 | rs9400701 | 110.63 | T/C | 0.4994 |
| 69 | 6 | rs1936797 | 119.32 | C/T | 0.4997 |
| 70 | 6 | rs12215895 | 132.9 | C/T | 0.4988 |
| 71 | 6 | rs9383760 | 152.27 | G/A | 0.5 |
| 72 | 7 | rs6967307 | 14.62 | G/A | 0.4984 |
| 73 | 7 | rs11975146 | 38.54 | C/T | 0.4984 |
| 74 | 7 | rs983399 | 63.36 | C/T | 0.4997 |
| 75 | 7 | rs10950240 | 76.42 | A/G | 0.499 |
| 76 | 7 | rs17165662 | 95.09 | C/T | 0.4995 |
| 77 | 7 | rs7794088 | 121.59 | C/A | 0.4989 |
| 78 | 7 | rs7806195 | 140.45 | C/T | 0.4981 |
| 79 | 7 | rs10248131 | 161.97 | T/C | 0.4989 |
| 80 | 8 | rs6559036 | 11.6 | C/G | 0.498 |
| 81 | 8 | rs13255913 | 31.53 | C/T | 0.4998 |
| 82 | 8 | rs9792122 | 52.7 | G/A | 0.4999 |
| 83 | 8 | rs9650306 | 61.83 | T/G | 0.4983 |
| 84 | 8 | rs7464123 | 73.29 | G/A | 0.4996 |
| 85 | 8 | rs9332439 | 88.67 | T/A | 0.4994 |
| 86 | 8 | rs7826497 | 104.25 | T/G | 0.4994 |
| 87 | 8 | rs7821151 | 124.86 | C/T | 0.4996 |
| 88 | 8 | rs4387006 | 145.08 | A/T | 0.4999 |
| 89 | 9 | rs2173709 | 18 | C/A | 0.4998 |
| 90 | 9 | rs1281350 | 37.27 | G/C | 0.4991 |
| 91 | 9 | rs2181926 | 52.4 | C/G | 0.4985 |
| 92 | 9 | rs7849506 | 71.17 | T/C | 0.4994 |
| 93 | 9 | rs1754073 | 88.62 | T/C | 0.4996 |
| 94 | 9 | rs687992 | 105.01 | A/G | 0.4998 |
| 95 | 9 | rs10818474 | 121.81 | T/C | 0.499 |
| 96 | 10 | rs10903579 | 2.72 | G/C | 0.4999 |
| 97 | 10 | rs12773782 | 45.22 | G/A | 0.4999 |
| 98 | 10 | rs10899912 | 58.96 | A/G | 0.4996 |
| 99 | 10 | rs1915624 | 75.59 | A/T | 0.4997 |
| 100 | 10 | rs4568951 | 95.95 | A/G | 0.5 |
| 101 | 10 | rs7923494 | 109.78 | G/A | 0.4998 |
| 102 | 10 | rs180695 | 124.23 | A/T | 0.4999 |
| 103 | 10 | rs7905678 | 155.09 | C/T | 0.4997 |
| 104 | 11 | rs6483705 | 32.98 | A/T | 0.4995 |
| 105 | 11 | rs1461905 | 53.36 | G/A | 0.4995 |
| 106 | 11 | rs4752917 | 60.33 | A/G | 0.4974 |
| 107 | 11 | rs4614474 | 81.84 | G/C | 0.4996 |
| 108 | 11 | rs11020453 | 89.51 | A/C | 0.498 |
| 109 | 11 | rs732930 | 106.18 | G/C | 0.4995 |
| 110 | 11 | rs10893026 | 123.83 | C/G | 0.4983 |
| 111 | 11 | rs1382498 | 146.06 | A/G | 0.4998 |
| 112 | 12 | rs11056314 | 29.5 | G/C | 0.4983 |
| 113 | 12 | rs10743629 | 47.1 | G/A | 0.4998 |
| 114 | 12 | rs4768795 | 56.86 | C/A | 0.5 |
| 115 | 12 | rs3842936 | 66.86 | A/G | 0.4994 |
| 116 | 12 | rs11180093 | 81.13 | C/T | 0.4995 |
| 117 | 12 | rs10858975 | 91.81 | T/A | 0.4999 |
| 118 | 12 | rs10860877 | 105.85 | C/T | 0.499 |
| 119 | 12 | rs4766748 | 118.63 | A/G | 0.4998 |
| 120 | 12 | rs1732453 | 137.22 | A/T | 0.4976 |
| 121 | 13 | rs2027576 | 16.1 | T/C | 0.4991 |
| 122 | 13 | rs9526313 | 43.84 | C/T | 0.4991 |
| 123 | 13 | rs3128050 | 52.91 | T/C | 0.499 |
| 124 | 13 | rs7327134 | 72.46 | G/A | 0.4998 |
| 125 | 13 | rs475580 | 82.36 | G/A | 0.4992 |
| 126 | 13 | rs1029204 | 105.02 | C/T | 0.4994 |
| 127 | 14 | rs11160611 | 15.44 | C/T | 0.4993 |
| 128 | 14 | rs2146228 | 34.42 | G/C | 0.4999 |
| 129 | 14 | rs17092583 | 47.51 | A/G | 0.5 |
| 130 | 14 | rs7152210 | 70.24 | G/A | 0.4992 |
| 131 | 15 | rs532707 | 34.61 | T/G | 0.499 |
| 132 | 15 | rs905451 | 72.2 | G/A | 0.5 |
| 133 | 15 | rs7162174 | 96.15 | T/C | 0.4999 |
| 134 | 16 | rs29572 | 17.42 | C/G | 0.4998 |
| 135 | 16 | rs4368165 | 35.3 | G/A | 0.4994 |
| 136 | 16 | rs2631806 | 70.48 | T/C | 0.4994 |
| 137 | 16 | rs12596410 | 81.22 | G/C | 0.4989 |
| 138 | 16 | rs3922878 | 114.1 | C/A | 0.5 |
| 139 | 17 | rs237348 | 28.72 | A/G | 0.4992 |
| 140 | 17 | rs4239211 | 42.41 | G/C | 0.4976 |
| 141 | 17 | rs4968414 | 76.5 | A/G | 0.4999 |
| 142 | 17 | rs11650378 | 98.14 | C/T | 0.4994 |
| 143 | 18 | rs11874322 | 33.19 | T/C | 0.4994 |
| 144 | 18 | rs7244025 | 40.44 | G/A | 0.4976 |
| 145 | 18 | rs508207 | 53.77 | A/G | 0.4979 |
| 146 | 18 | rs1451940 | 67.91 | T/C | 0.499 |
| 147 | 18 | rs2406342 | 102.95 | G/T | 0.4999 |
| 148 | 19 | rs6510799 | 0.97 | T/G | 0.4998 |
| 149 | 19 | rs12974188 | 47.89 | C/T | 0.498 |
| 150 | 19 | rs8109462 | 67.02 | T/C | 0.4983 |
| 151 | 19 | rs2058318 | 97.06 | G/C | 0.4995 |
| 152 | 20 | rs3904872 | 11.03 | A/G | 0.4995 |
| 153 | 20 | rs10854249 | 43.03 | A/C | 0.4999 |
| 154 | 20 | rs221308 | 48.31 | T/C | 0.498 |
| 155 | 20 | rs3092349 | 61.25 | A/G | 0.4994 |
| 156 | 20 | rs6071469 | 89.9 | G/T | 0.4997 |
| 157 | 21 | rs2822967 | 2.01 | G/T | 0.4977 |
| 158 | 21 | rs12626903 | 26.17 | A/C | 0.4995 |
| 159 | 22 | rs5992587 | 0.15 | G/A | 0.4985 |
| 160 | 22 | rs135139 | 48.93 | C/A | 0.4999 |

Supplementary Table 1 Information of finally selected SNPs for paternity testing of Koreans
